# Collaboration in the return-to-work process after sick leave due to common mental disorders: a qualitative study of stakeholders’ views on goals and roles

**DOI:** 10.1186/s12889-024-19063-y

**Published:** 2024-06-11

**Authors:** Veronica Svärd, Zana Arapovic Johansson, Lisa Holmlund, Therese Hellman, Lydia Kwak, Elisabeth Björk Brämberg

**Affiliations:** 1 Department of Social Work, School of Social Sciences, Södertörn University, Huddinge, SE-141 89 Sweden; 2 Division of Insurance Medicine, Department of Clinical Neuroscience, Karolinska Institutet, Stockholm, SE-171 77 Sweden; 3 Unit of Intervention and Implementation Research for Worker Health, Institute of Environmental Medicine, Karolinska Institutet, Stockholm, SE-171 77 Sweden; 4 Division of Occupational Therapy, Department of Neurobiology, Care Sciences and Society, Karolinska Institutet, Huddinge, SE-141 83 Sweden; 5 Occupational and Environmental Medicine, Department of Medical Sciences, Uppsala University, Uppsala, SE-751 85 Sweden; 6 Department of Occupational and Environmental Medicine, Uppsala University Hospital, Uppsala, SE-751 85 Sweden

**Keywords:** Return to work, Rehabilitation, Collaboration, Coordination, Goal, Role, Responsibility, Common mental disorders, Sick leave

## Abstract

**Background:**

This study explores how the goals of collaboration in the return-to-work (RTW) process for people with common mental disorders are described by the stakeholders involved, and how they experience stakeholders’ roles and responsibilities in relation to these goals.

**Methods:**

Interviews were conducted with 41 participants from three Swedish regions. Nine of the participants were workers, six employer representatives, four occupational health professionals, four social insurance officers, 18 RTW coordinators and five physicians. Thematic analysis was conducted.

**Results:**

Three main themes and overarching goals when collaborating on RTW were identified. In the first theme, ‘creating an informative environment’, all stakeholders emphasised clear roles and responsibilities. The second theme, ‘striving for consensus in an environment of negotiations’, addressed negotiations about when and how to collaborate, on what and with whom, and reveal different views on stakeholders’ goals, roles and responsibilities in collaboration. The third theme identified goals for ‘creating a supportive environment’ for both workers and other stakeholders. Coordinators are found to have an important role in achieving a supportive environment, and in neutralising power imbalances between workers and their employers and social insurance officers.

**Conclusions:**

Competing goals and priorities were identified as hindering successful collaboration, contributing to a spectrum of complex versus easy RTW collaboration. This study suggests some basic conditions for achieving a collaborative arena that is neutral in terms of power balance, where all stakeholders can share their views.

## Background

Various stakeholders collaborate in the return-to-work (RTW) process for people on sick leave due to common mental disorders, i.e., depression, anxiety, adjustment disorders and stress-related mental disorders. The stakeholders (e.g., employers, occupational health services, the Swedish Social Insurance Agency (SSIA) or social services) often represent organisations that are characterised by different regulations and guiding principles – and except for the shared overall goal of the worker’s return to work, there is not always a consensus on the goals of RTW collaboration [1–3].

There is a consensus that the level of collaboration between stakeholders influences the successful management of long-term sick leave [4], and some weak evidence that enhanced communication and collaboration reduce sick leave [5]. Moreover, different perspectives, priorities and goals among stakeholders could hinder the RTW process for people with common mental disorders, and cause confusion and uncertainty about how and when to return to work [6].

While there is a relatively large amount of research observing stakeholders’ different perspectives and goals of the RTW process [4, 7], and how this hinders the collaboration between them [8–11], there is less research on these stakeholders’ views on the goals of RTW collaboration itself. Research shows, however, that interdisciplinary collaboration regarding rehabilitation of people with common mental disorders is facilitated when stakeholders share information, are proactive and flexible, respond promptly, and agree on the overall goal of RTW [12]. Moreover, trust and respect between the parties seem to be essential for clear and supportive communication [12, 13]. Clearly defined roles, expectations and goals facilitate the teamwork required in RTW planning and improve the collaborative process [14]. To alleviate the confusion regarding the stakeholders’ roles in the RTW process, Corbière et al.’s [2] scoping review offers a detailed role description for eleven different stakeholders at different phases of the RTW process. However, even with the roles clarified, the authors found that stakeholders’ different goals and agendas for collaborating may still be unclear or in conflict.

To promote RTW, support the person on sick leave, and facilitate RTW collaboration, RTW coordinators (RTWCs) have been implemented in many countries [6, 15–19]. While some reviews have found that RTW coordination has no effect on the RTW rate for people with common mental disorders on sick leave [15, 16], others show moderate evidence that RTW coordination results in a higher probability of RTW for a broad spectrum of disorders and injuries [17, 18, 20]. However, the RTWCs’ roles, strategies and responsibilities vary considerably [9, 15, 21, 22], depending on which sector they are employed in, country-specific legislation, the social insurance system and the healthcare system. In 2020, the Swedish healthcare services became obliged to offer sick-listed patients coordination of the rehabilitation and RTW process if needed. Previous studies conducted in Sweden have found that RTWCs’ involvement enhances communication and collaboration between the stakeholders involved in the RTW process [11, 23–25], and provides encouragement and structure for the patient’s RTW process [23–26]. However, these studies show different views on RTWCs’ assignments and roles.

While it is generally understood that the stakeholders involved in the RTW process share a common goal of promoting RTW, and that they should collaborate to achieve this, it is under-explored what goals they have for such collaboration, and what roles and responsibilities they perceive themselves to have in relation to such goals. Different stakeholders can, for example, have different expectations of the specific goals of a collaboration meeting and what it should result in, besides a general expectation of promoting the RTW process. The present study aims to explore how the goals of the collaboration in the RTW process for people with common mental disorders are described by the stakeholders involved, and how they experience their own and other stakeholders’ roles and responsibilities in relation to the goals. Additionally, we explore what shared and competing goals of the collaboration are expressed by the stakeholders.

## Theoretical framework

Although stakeholders may share the overall goal of the worker achieving a safe, timely and sustainable RTW, Young et al. [3] show that stakeholders from different organisations sometimes regard the efficiency of RTW (i.e., sparse use of resources in the RTW process) to be more or less important than the effectiveness (i.e., achieve RTW). This might partly explain why work resumption is not achieved, although it appears to be a ‘win-win’ situation for all involved. The organisations have different goals, restrictions and abilities, which may affect their priorities in RTW collaboration, and thereby their ‘net benefit’ from RTW [3]. Stakeholders are commonly instructed to share the organisational goals, rules and decisions. Those in positions of authority have the right to negotiate and make decisions. Ahrne [27] points out that a representative without such a mandate may face difficulties in upholding trustful relationships with stakeholders outside their organisation; trust in the person does not necessarily mean that you can trust what the organisation will decide. To enable shared goals and collective decisions, all representatives must participate in collaboration, share information and negotiate, otherwise the outcome may be unexpected [27].

Organisations often collaborate to achieve common goals that are outside their own control, as in the case of the RTW process. Collaboration involves a “negotiated environment” [26], where representatives strive to gain benefits depending on their own organisations’ goals. This includes negotiating boundaries for what they should do, and what they claim other stakeholders should do [21, 28]. However, negotiation is only possible when the stakeholders have a mutual interest in stabilising their interaction and overcoming imbalances in influence and power relations through discussions and agreements [27].

However, it is often the organisation that determines, e.g., whom to help, whom to collaborate with, what needs to be done and by whom. This can lead to inflexibility for representatives in external collaboration. Without the flexibility to negotiate, an organisation’s representative can bring power to the negotiation, e.g., warn that the representative cannot take responsibility for how other stakeholders will (re)act, or warn about what might happen. As negotiations imply uncertainty, an organisation sometimes strives to show a united face to keep strength in the negotiation. Aiming to break this unity, other stakeholders may try to appeal to the representative’s more human and softer side [27] – a tendency seen in a previous study about RTW coordination and collaboration [21].

## Methods

This study used a qualitative approach involving semi-structured individual interviews and a focus group, analysed thematically [29]. The study was inspired by a phenomenological approach as the focus was to identify the various stakeholders’ experiences, perspectives, and behaviours in the context of RTW collaboration [30].

The overall project was described in a study protocol [31]. Results about facilitators and barriers to the coordination of RTW for workers on sick leave due to common mental disorders [11], as well as ethical issues that arise in the coordination of RTW [32], have been reported. Ethical approval was obtained from the Regional Ethical Review Board in Stockholm (Dnr 2018/677 − 31/2; 2018/2119-32). The reporting follows consolidated criteria for reporting qualitative research (COREQ) [33].

### Participants

The participants were individuals with ongoing or previous sick leave due to common mental disorders and stakeholders who had participated in collaboration meetings with such individuals (see study protocol for more details about the inclusion/exclusion criteria and the recruitment process [31]). The data collection took place in three Swedish regions: Västra Götaland, Uppsala, and Stockholm. Individual interviews were conducted with 41 participants: nine workers, six employer representatives (ERs) (two chief executive officers and four HR specialists), four occupational health professionals (OHP), four officers at the Swedish Social Insurance Agency (SSIA) and 18 RTWCs (Table 1). A focus group interview was conducted with five GPs working at different primary healthcare centres.

The SSIA officers worked with insured people before 90 sick-leave days and one with unemployed people on long-term sick leave. The OHPs were employed by occupational health services or by large employers. The RTWCs worked at primary healthcare centres in three regions, and the GPs worked at different primary healthcare centres in one region. Among the workers, four worked in the public sector and five worked in the private sector. Among ERs, four worked in the public sector and two worked in the private sector.

**Table 1:** Participants’ characteristics and type of interview

Participants	Number	Organisation	Gender *(female / male)*	Professional experience *(years)*	Type of interview *(face-to-face / telephone / focus group)*
Return-to-work coordinators (RTWCs)	18	PHC	18/0	1–8	15/3/0
General practitioners (GPs)	5	PHC	2/3	2–30	0/0/1
Workers on sick leave	9		8/1	≤ 4^1^	2/7/0
Employer representatives (ERs)	6		4/2	2–30	0/6/0
Occupational healthcare professionals (OHPs)	4	OHS	4/0	4–18	3/1/0
Swedish Social Insurance Agency (SSIA) officers	4	SSIA	2/2	2–5^2^	3/1/0
Total	46		38/8	-	23/18/1

PHC = Primary healthcare centre, OHS = Occupational Health Service

^1^ At the current workplace

^2^ Data is missing for one SSIA officer

### Data collection

The interviews were conducted by the fourth and the sixth author between June 2018 and December 2019, and a strategic sampling procedure was used. The recruitment process began with the fourth and the sixth author informing primary healthcare centre managers and RTWC process leaders in the three regions about the study and asking them to invite RTWCs and GPs. The RTWCs then forwarded information about the study and invitations to the employers and the workers, who contacted the research team if they were interested in participating or having more information about the study. The OHPs and SSIA officers were selected by their organisations after the research team sent information and invitations to their managers. Semi-structured individual interviews were held by telephone [34] or face-to-face at a location chosen by the participants (Table 1). The intention was to conduct individual interviews with GPs. However, the primary healthcare centre managers requested that the GPs should be interviewed in a focus group, instead of individually, based on the argument that it would be favourable for GPs to discuss these issues with other GPs and learn from each other and less time-consuming since it could fit in a collegial meeting. As the participation of GPs was important, researchers agreed on this change. The interviews lasted 20–60 min, and the focus group lasted 90 min.

Three similar interview guides were designed by the research team: one for workers, one for RTW professionals (RTWCs, GPs, OHPs and SSIA officers) and one for ERs. The first part of the interview guides comprised open-ended questions about barriers and facilitators relating to the RTW collaboration and coordination, with questions developed from the Consolidated Framework for Implementation Research (https://cfirguide.org/). The second part comprised open-ended questions about the ethical aspects of RTW coordination. Results regarding the ethical aspects of RWT coordination are published elsewhere [32].

All participants were asked: *“In your opinion, what is coordination aiming at?”*, and questions about their role in coordination, issues that have not been the focus of previous analysis [11, 31]. Interview questions were for example: (workers) *“Can you tell me about the coordination you have had with your employer and the primary healthcare?”*; (managers) *“How did you experience taking part in the coordination?”*; (representatives from the primary healthcare centre, SSIA, and OHS) *“Can you tell me how you have formed your work with coordination, and your reasons for forming the coordination in such way?”* The full interview guides are available in the study protocol [31].

Notes were taken during the interviews, and at the end of the interview session, the notes were checked with the interviewee to confirm the accuracy. All participants were offered the opportunity to review their interview transcripts, and none of the participants who received their transcripts suggested any changes.

### Data analysis

Secondary analysis [30, 35] was carried out on rich interview data. There were no specific interview questions about the goals of collaboration; instead, this was something that was often brought up by the participants themselves as a response to the open-ended interview questions. All interviews were transcribed verbatim, and abductive thematic analysis was used to identify patterns in the data and generate themes [29]. The coding process started as a form of codebook thematic analysis [30, 36], in terms of inductive structured coding for identifying segments of relevance for the research aims, and for documenting the analysis. In this early phase, preliminary themes were identified and in the later phases, the coding process was characterised by codes evolving in an organic way, where some shifted and changed as they were refined or collapsed with other codes [30]. The analysis was inspired by Braun and Clarke’s six-phase process for data engagement, coding and theme development [29]. In the first phase, the first and the second author read through the transcriptions separately to get familiar with the data and generated an initial coding of data segments relating to the aim of the study. Because the data showed overlap between ‘goals of rehabilitation’ and ‘goals of collaboration’, it was carefully reviewed in a second phase, where the first author removed segments regarding goals of rehabilitation. After revision, the dataset included segments that explicitly addressed the study’s aims, e.g., *“My role in meetings is to facilitate communication”*, as well as segments providing latent descriptions, such as *“It was good to meet to be able to share information and clarify the situation”*, illustrating two goals of collaboration meetings. Based on the preliminary codes and the segments included, the first and the second author conducted a third phase of the analysis, by reviewing the codes and organising them into two broader dimensions: shared and competing goals, and roles and responsibilities in RTW collaboration. In the first three phases, several preliminary themes were identified, such as ‘the importance of information’, ‘role clarity’, ‘hindering goal conflicts’, ‘negotiations in collaborative meetings’, ‘striving for consensus’, ‘power imbalances’ and ‘supportive environment’. In discussing preliminary themes during the third phase, central concepts such as collaboration, negotiation and power imbalance were identified as important theoretically informed concepts for understanding the findings and how themes related to each other. In the fourth phase, while reviewing the themes, the first author conducted a more detailed abductive thematic analysis exploring inter-relationships between theoretical concepts, themes and sub-themes, and this resulted in three suggested main themes, and several sub-themes. In the fifth phase, the suggested themes and sub-themes were critically discussed, refined and renamed by the first, second, third and the sixth author, resulting in the final three main themes – ‘creating an informative environment’, ‘striving for consensus in an environment of negotiations’ and ‘creating a supportive environment’ – with underlying sub-themes (see Tables 2 and 3). The first, second, third and the sixth author critically discussed the coding and the theoretical conceptualisations during the analysis process until a final consensus was reached. In the sixth and last phases, fourth and the fifth author reviewed the suggested analysis and clarifications were made for reporting the results.

**Table 2:** The shared and competing goals of collaboration in the RTW process as expressed by different stakeholders

Themes and sub-themes	Shared goal	Stakeholders that expressed the shared goal	Competing goal	Stakeholders that expressed the competing goal
**Creating an informative environment**				
	Share information	W, RTWC, GP	Worker’s integrity	W, SSIA
	Improve communication	W, RTWC, SSIA, ER, OHP	Withholding information from employer or SSIA	RTWC, GP, SSIA, ER, OHP
	Clarify regulations, responsibilities and expectations	W, SSIA, ER		
	Achieve an open and honest conversation	W, RTWC, GP, ER		
**Striving for a negotiative environment**				
When to collaborate	Early collaboration to decrease sick leave length and prevent long-term sick leave	RTWC, GP, SSIA, ER, OHP	Needs for collaboration on long-term sick leave cases	GP
	Sustainable and timely RTW (not too early or late)	RTWC	Negative beliefs about collaboration for patients on long-term sick leave	RTWC
			SSIA only collaborates late in the RTW process	RTWC, GP, SSIA
			Employers collaborate only when worker is fully recovered	RTWC, GP, SSIA
What to collaborate on, and with whom	Clarify the worker’s situation	W, RTWC	A different activity or job, instead of RTW	W, RTWC, GP
	Improve rehabilitation and speed up the RTW process	RTWC, ER, OHP	Other priorities due to worker’s home situation	W, RTWC, SSIA, ER
	Facilitate and put the RTW plan into practice, evaluations and follow-ups of RTW plan	W, RTWC, ER, OHP	Stakeholders focusing on their own priorities, rather than shared goals	W, RTWC, GP, SSIA, ER, OHP
	Find agreements and consensus	W, RTWC, SSIA, ER, OHP	RTWC not wanting to collaborate on certain diagnoses	RTWC
	Find shared and forward-looking goals	W, RTWC, SSIA	Worker being too sick for RTW collaboration; the focus must be on treatment	SSIA
	Solve competing goals and barriers	W, RTWC, SSIA	Employer not wanting worker to return to work	SSIA
Forms of collaboration	Meetings create links between stakeholders	W	Stakeholders avoiding collaboration, focusing on their own priorities	RTWC, GP, SSIA, ER
	Create structure in collaboration	W, RTWC	Stakeholders interpreting concepts for collaborating meetings differently	RTWC
**Creating a supportive environment**				
	Offload and provide worker with emotional support, empathy, hope and motivation	W, RTWC, SSIA, OHP	GPs protecting the worker from the RTW process	GP, SSIA, ER, OHP
	Worker being heard, prioritised and actively involved, promote power balance	W, RTWC, ER, OHP	Prioritise the work environment for the worker’s colleagues	RTWC
	Provide stakeholders with support and a sense of security	W, GP, RTWC, ER		
	Jointly handle the situation, work together and share responsibility	W, RTWC, GP, ER, OHP		

W = worker; GP = general practitioner; RTWC = return-to-work coordinator; SSIA = Swedish Social Insurance Agency officer; ER = employer representative; OHP = occupational health professional; RTW = return-to-work

**Table 3:** The roles and responsibilities in RTW collaboration as expressed by different stakeholders

Themes and sub-themes	Worker	Return-to-work coordinator	General practitioner	SSIA officer	Employer representative	Occupational health professional
**Informative environment**						
	Clear roles and responsibilities are needed (RTWC, SSIA, ER, OHP)	Clear roles and responsibilities are needed (RTWC, SSIA, ER, OHP)	Clear roles and responsibilities are needed (RTWC, SSIA, ER, OHP)	Clear roles and responsibilities are needed (RTWC, SSIA, ER, OHP)	Clear roles and responsibilities are needed (RTWC, SSIA, ER, OHP)	Clear roles and responsibilities are needed (RTWC, SSIA, ER, OHP)
	Should take an active role in RTW collaboration (GP, ER)	Transfer information and ask the right questions (W, RTWC)		A mediator, explaining the legal system (SSIA)	Fulfilling role and responsibilities require information about worker’s diagnosis (W) and functions (OHP)	
		An informative role (GP, SSIA), having knowledge about the societal system and all stakeholders’ roles (RTWC, SSIA)				
**Negotiative environment**						
When to collaborate		Should focus on those not yet on sick leave (SSIA), or early in the sick leave process (RTWC), or regardless of the length of sick leave (RTWC)		Only later when worker has become healthy enough to RTW, or when others do not take responsibility (SSIA)		
		Needed in long-term sick leave cases (GP)				
What to collaborate on and with whom	Should focus on the future and work abilities (SSIA)	Map worker’s situation (RTWC)	Should confront worker regarding a new workplace, rather than sickness absence (GP)	Should focus on medical status and treatment (SSIA)	Ensuring there is a shared goal among stakeholders (ER)	
		Should have *or not* have a role in socially complex cases, worker-employer conflicts, or where there is no employer (RTWC)	Should take their responsibility to write sick leave certificates (OHP)			
Forms of collaboration		Provide structure by actively coordinating contacts, meetings and follow-ups (W, RTWC, SSIA, ER)	Often exchanged, hard to reach and avoiding contact (W, RTWC, SSIA, ER, OHP)	Should have a more collaborative role, attending meetings (RTWC, GP, OHP)		Active role, initiate contacts, give advice and recommendations (OHP)
		Laid-back and consultative role (RTWC)	One GP only should be responsible for new sick leave cases at PHC (GP)			Laid-back role, asking questions (OHP)
		The link between stakeholders (W, ER, SSIA)				
**Supportive environment**						
	Work together and share responsibility (W, ER, OHP, GP, RTWC)	Work together and share responsibility (W, RTWC, GP, ER, OHP)	Work together and share responsibility (W, RTWC, GP, ER, OHP)	Work together and share responsibility (W, RTWC, GP, ER, OHP)	Work together and share responsibility (W, RTWC, GP, ER, OHP)	Work together and share responsibility (W, RTWC, GP, ER, OHP)
		A neutral (W, RTWC, SSIA, ER), therapeutic or advocating role for worker (W, RTWC, ER)	Inherent role conflicts (GP)		The Human Relations Specialist should have a neutral role (ER)	A neutral role, supporting both employer and worker (OHP)
		Important role for worker’s trust in stakeholders (W, RTWC)	Stand by the worker (GP, SSIA)		The employer should provide necessary support and work adjustments (W, RTWC, SSIA, ER)	Not being on the employer’s side (OHP)
		Promoting power balance (RTWC)	Provide employer with a sense of security regarding how to act (GP)			Being on the employer’s side, being tough at meetings (OHP)
		Offloading GP’s role in collaboration (RTWC, GP, SSIA)				
		Support managers’ decision-making (ER)				

W = worker; GP = general practitioner; RTWC = return-to-work coordinator; SSIA = Swedish Social Insurance Agency officer; ER = employer representative; OHP = occupational health professional; RTW = return-to-work; PHC = Primary healthcare centre

### The Swedish sickness insurance model

In Sweden, all workers with income from work, unemployment or parental benefits can be granted full- or part-time sick leave benefits up to 80% of lost income from the SSIA if they have reduced work capacity due to illness or injury. The SSIA assesses the eligibility for benefits according to the so-called ‘rehabilitation chain’, which means that individuals’ work ability is assessed in increasingly broader terms as time passes. When the study’s data collection took place, work ability was assessed for any job on the labour market after 180 sick-leave days. After 365 days, benefits are only granted in cases of severe illness.

The SSIA is responsible for coordinating the rehabilitation process, although it cannot require other stakeholders to carry out rehabilitation measures. Healthcare is responsible for medical treatment and rehabilitation, and physicians issue sick leave certificates, although they are not obliged to. Employers are obliged to establish a RTW plan, taking measures for rehabilitation and making job modifications [37].

### Stakeholder collaboration around a person on sick leave

Stakeholder collaboration can take different forms around a person on sick leave, as individual or group contacts via mail, or as online or face-to-face meetings. For workers and employers, especially, it is not well-known that some stakeholder meetings have different terminology and meanings, so the current study is not limited to the experiences of certain types of collaboration meetings, or types of contacts between stakeholders. There are, however, two types of collaboration meetings that often occur, online or face-to-face: ‘three-party meetings’ and ‘reconciliation meetings.’ [38] A three-party meeting often occurs early in the workers’ sick leave and focuses on the work situation. Participants are usually the worker and representatives from healthcare, and the employer/Employment Service/social services (depending on the person’s work status). The other type, ‘reconciliation meeting’, is often initiated by the SSIA later in the workers’ sick leave and aims to clarify and coordinate the measures the patient needs to be able return to work (medical, social or labour market measures). A reconciliation meeting involves the SSIA, the worker and at least one other stakeholder that can influence the patient’s situation [38]. This could be e.g. a GP, RTWC, ER, OHP and/or a representative from the Employment Service, or a trade union.

## Results

The results show that there were shared goals of RTW collaboration among stakeholders which facilitate collaboration, but there were also competing goals that prevent successful collaboration. When addressing goals of collaboration, as well as roles and responsibilities, the participants generally referred to different aspects of the collaborative environment facilitating or hindering RTW collaboration. The analysis identified shared and competing goals and perceived roles and responsibilities relating to three main themes when collaborating around RTW: creating an informative environment, striving for consensus in an environment of negotiations and creating a supportive environment. The three themes reflect the overall goals of collaboration, which include underlying sub-themes and codes of different shared and competing goals (Table 2), and perceived roles and responsibilities (Table 3).

### Creating an informative environment

In the first theme, creating an informative environment, participants emphasised that collaboration should occur in an environment where information can be shared between the stakeholders. The participants formulated different goals concerning this theme, and elaborated on stakeholders’ roles and responsibilities that promoted or obstructed the creation of a joint informative environment.

#### Shared and competing goals of collaboration

The collaboration meetings were described as being central to the shared goal of creating an informative environment (Table 2). Collaboration meetings enabled the stakeholders to have their say and share information. Other important goals of collaboration were to improve the communication between the involved stakeholders and clarify regulations and expectations to avoid or sort out misunderstandings. One SSIA officer who worked with insured people before 90 sick leave days said:

Sometimes, when I go to a collaboration meeting, I try to find out what abilities the insured individual has, not with the intention of saying “No, you’re not entitled to sick leave benefit now”, that’s not the purpose I think, but the abilities if you’ve someone who may have a little low self-esteem or something, trying to bring up “But you can do this and that”, and lifting them to get over this threshold and get back. And sometimes I experience that some care providers, even coordinators, may oppose that, thinking that’s for the purpose of saying no to benefits. It has happened. It’s not good really and I try, everyone is there for one and the same purpose, but we speak different languages.

Several stakeholders described that the goal of collaborative meetings was to have an open and honest conversation leading to a shared understanding of the worker’s situation, problems and needs. Therefore, being honest with their goal was essential to facilitate an open, informative environment.

The stakeholders described workers hesitating to share information with the employer for privacy reasons or due to worker-employer conflicts. One SSIA officer stated that although the worker’s privacy cannot be questioned, it can affect collaboration when the employer does not obtain important information about the illness. Some GPs were described as withholding facts from the SSIA due to the risk of patients being denied sick leave benefits. GPs themselves discussed difficulties knowing whether a patient has hidden goals or circumstances for not returning to work. However, while some workers avoided addressing private issues during collaboration, others found them essential to discuss when planning work adjustments.

#### Roles and responsibilities

Central to creating an informative environment were clear roles and responsibilities during the RTW collaboration (Table 3). The RTWCs emphasised the importance of everyone knowing each other’s assignments and being committed to shared responsibility in the collaboration. The ERs’ opportunities to understand the nature of workers’ sick leave and being sensitive to their needs were stressed as important for managers’ possibilities of fulfilling their responsibilities for the RTW process. On the one hand, ERs and GPs said workers were responsible for actively participating in RTW collaboration, informing them about their current situation and needs. On the other hand, workers mostly talked about the RTWCs’ role, describing them as transferring information and asking the right questions.

SSIA officers also argued that the RTWCs should have an informative role, being knowledgeable about the societal system, stakeholders’ roles and interventions. The SSIA officers described themselves as mediators, ensuring that the stakeholders communicate. Their role included explaining the regulations in simple terms, and one said:

It’s important that they know my role. Otherwise, they can set up against me, not listening when I say that we should take the next step towards RTW.

When everyone had reasonable expectations of what the SSIA can and cannot do, the collaboration was described as turning out well.

### Striving for consensus in an environment of negotiations

The second theme, striving for consensus in an environment of negotiations, pertained to an environment involving negotiations about goals, roles and responsibilities regarding the conditions of when to collaborate, on what, with whom and how – and the strive for consensus regarding these conditions. Subsequently, three sub-themes were identified: when to collaborate, what to collaborate on and with whom, and forms of collaboration.

#### When to collaborate

##### Shared and competing goals of collaboration

Most participants shared the goal of early RTW collaboration. However, there was no consensus on what “early” meant. The SSIA officers emphasised collaboration between the worker and employer early in the RTW process, but did not share the goal that *all* stakeholders must collaborate early on. The SSIA officers said that they should be involved later in the process, an approach that others found frustrating and tried to negotiate on. When discussing common mental disorder cases, one HR manager proposed clear demands on early collaboration and said: *“I’d like that there to even be… maybe even legislated that on day one [of sickness], you come together.”*

Negotiations about when to collaborate took place between several stakeholders. The GPs said they mostly participated in collaborative meetings regarding long-term sick leave cases. This did not harmonise with the RTWCs’ overall goal of preventing long-term sick leave, which involved prioritising collaboration on workers with shorter sick-leave periods. Several participants further described putting pressure on employers to collaborate, i.e., employers who did not want the worker back until they were fully recovered, and thus lacked an interest in the goal of early collaboration.

##### Roles and responsibilities

The RTWCs had different opinions regarding their role and when to collaborate. Some refused to coordinate workers who did not progress in the RTW process and prioritised those on sick leave for less than three months. Other RTWCs rejected this approach and claimed that the GPs’ role should be to refer all workers in need of RTW support.

The SSIA officers said their role was to attend meetings only when others did not take responsibility, as they had a responsibility to negotiate and solve such barriers. However, they viewed their own role differently depending on their target group of insured workers. One who worked with long-term sick leave said that a criterion for attending a meeting was that the worker has become healthy enough to gradually return to work.

#### What to collaborate on and with whom

##### Shared and competing goals of collaboration

An environment of negotiation also pertained to a context in which a range of different goals regarding what to collaborate on and with whom were included. From the worker’s point of view, one goal was to clarify and reach a consensus about their current situation, including their needs and the barriers and possibilities for returning to work. Several stakeholders had a goal of improving the quality of rehabilitation and speeding up the RTW process. Negotiating what to collaborate on was often expressed as finding agreements and consensus about goals related to the rehabilitation and RTW plan. This concerned, e.g., work adjustments, problem-solving in the workplace, writing a RTW plan, and evaluating and following up on this plan. One OHP said that having more competencies participating in collaboration made it easier to agree on a sustainable plan, and continued:

When we do things together and jointly, everyone hears the same thing, working towards a common goal, then we get there too. And you minimise these bumps in the road.

Another goal with collaboration was to reach a consensus about forward-looking goals. Most RTWCs stressed that setting goals should focus on the future, providing the worker with a feeling that all stakeholders are collaborating around and striving towards a common goal. However, the RTWCs’ goals differed. While some focused solely on returning to work, others talked about broader goals involving improved health, quality of life and activities that break destructive sick leave patterns.

A general goal with collaborative meetings was to negotiate on and resolve conflicting or competing goals. Clarifying whether the worker’s goal is to stay at their workplace, find a new job, engage in education or not work at all was deemed crucial for successful collaboration. One RTWC elaborated on various reasons for workers not sharing the goal of returning to work:

It may be that you don’t want to be at work because you want to be at home, or that you need to stay at home if you’ve relatives who are ill, or whatever the case may be. Or it could be that things aren’t good at work, you’ve a bad relationship with your manager or a conflict with your manager or colleagues, or you’ve a job you really don’t like. You want to change jobs.

Another shared goal of collaboration among many stakeholders was to focus on present symptoms and needs, and to avoid dwelling on possible causes behind the sick leave. However, several workers described their home situation as causing their illness; thus, managing their home situation, rather than RTW, became their primary goal. Other stakeholders agreed that social difficulties in the worker’s private life had a negative effect on the shared goal of RTW, as they impeded the possibility of rehabilitation. Some RTWCs said social difficulties, such as family conflicts, or spending a lot of time taking care of children with neuropsychiatric disorders or other sick relatives, must be dealt with before rehabilitation can begin. By contrast, others questioned the right to sick leave benefits, and thus collaboration taking place at all, if social difficulties were causing an inability to work.

Other competing goals regarding what collaboration should deal with were described by OHPs and ERs as GPs focusing on sickness and barriers rather than possibilities, the SSIA officers focusing on work abilities and ending the sick leave benefit, and the employer getting caught in the middle. Negotiations took place on whether collaboration should focus on a sustainable RTW or getting workers back at work before day 180.

The most prominent competing goal highlighted by GPs was workers attending a primary healthcare centre with sick leave rather than RTW in mind, arguing that this hindered rehabilitation and recovery.

##### Roles and responsibilities

The RTWCs described their role as mapping the worker’s situation, and identifying problems and solutions. There was no consensus, however, regarding *in which cases* they should have a role. Some prioritised workers who wanted to return to their current workplace, while others also coordinated workers with new education or a new job as the goal. Some refused cases with an ongoing worker-employer conflict or when social difficulties caused the sick leave. By contrast, others said they had an essential role in helping workers to manage social problems that obstructed rehabilitation.

The SSIA officers described their role at meetings as focusing on the worker’s medical status, functions, limitations and treatments. They said the workers were responsible for articulating their work abilities and thinking about the future. Similarly, one GP described the GP role as confronting workers who wanted to stay on sick leave when there is no medical reason for this, and rather encouraging the worker to seek a new workplace or alternative solutions if they could not work or hesitated to work.

One ER described their role as ensuring that there is a shared goal. Some RTWCs stated, however, that employers can too easily escape their responsibilities in the rehabilitation and RTW process (see Table 3).

#### Forms of collaboration

##### Shared and competing goals of collaboration

There was a broad consensus that RTW collaboration should take the form of joint meetings between the stakeholders involved, with the goal of finding a structure for both collaboration and the RTW process. RTWCs found collaborative meetings at the worker’s workplace to be favourable, as this helped to play down feelings of anxiety or fear of work.

Participants described competing goals as involving stakeholders from the different organisations working in separate silos. GPs and SSIA officers in particular were described as having priorities other than responding to contact attempts and participating in meetings. RTWCs explained how stakeholders interpreted concepts for collaborative meetings differently, e.g., what ‘reconciliation meeting’ means. Such differences contributed to unclear goals for meetings, who was expected to attend, or in what phase of the RTW process the meetings should take place.

##### Roles and responsibilities

The RTWCs were described as the driving force in order to reach consensus, taking an active role in booking and leading meetings, clarifying aims, and providing structure, follow-ups and continuity in collaboration. However, some RTWCs said they took a laid-back and consultative role, providing assessments to GPs, and encouraging others to make contact. RTWCs were also described as having a linking role, and filling the gap of non-participating GPs.

Many pictured GPs as hard to reach, being constantly replaced and failing to meet their responsibilities to follow up on medical treatment. This made compliance with the rehabilitation plan and rehabilitation chain impossible. The OHPs appreciated having direct contact with RTWCs to facilitate collaboration with primary healthcare centres. Due to limited time and possibilities to participate actively in collaboration, the GPs negotiated how to delimit or clarify their roles. To prevent patients having their GPs replaced with others, one primary healthcare centre had appointed one GP to handle all new sick leave cases and contacts with the SSIA, while the RTWC stayed in contact with the employer. The GPs generally found it difficult when SSIA officers wanted direct contact with the GP, and believed RTWCs were better suited to coordinate the contacts.

While SSIA officers described themselves as having a collaborative responsibility, other stakeholders argued they took too limited a role in collaboration. One OHP stressed the importance of SSIA officers attending meetings because of their legal mandate to decide on sick leave benefits and work training, and said *“Unfortunately, they cannot really live up to this as things are at present, and haven’t been able to for a couple of years”*, referring to the fact that they had been instructed not to prioritise meetings.

While some OHPs pictured their role as being active in meetings, initiating contact on behalf of the employer, or giving advice and recommendations, other OHPs took a more laid-back role and focused on the process in order to proceed.

### Creating a supportive environment

In the third theme, creating a supportive environment, the findings show goals, roles and responsibilities that create or impede a supportive environment for the worker and other stakeholders. Further examples of how a supportive environment can be achieved in collaboration are also described in previous sub-themes, such as clear communication, structure and roles, information sharing and problem-solving.

#### Shared and competing goals of collaboration

Several stakeholders expressed a need for a sustainable RTW pace that did not cause problems in terms of recurrent sick leave, and many stressed that a shared goal of collaboration was to create a supportive environment to achieve this. This involved a sense of security about how to act for RTW, especially for managers and workers. Both RTWCs and SSIA officers described goals as focusing on abilities, possibilities and strengthening the worker’s mental health, self-esteem and self-confidence to manage RTW. In line with this, shared goals were offloading and providing the worker with emotional support, and expressing empathy, goodwill, hope, positivity and motivation. One worker said:

I feel that they’re a team and that they’ve a similar idea of what the goal is. Because I can imagine that it could’ve been quite disjointed otherwise. […] It feels like they’ve laid some kind of good foundation and that we’re moving towards a goal [of returning to work] together in a way, with the same method and ambition.

To avoid workers being passed between stakeholders, having the worker as the priority at collaborative meetings was emphasised, and one worker described such meetings as *“it helps enormously to have a… what would you call it, a line of defence.”*

Referring to power imbalances between employers and workers, one OHP stressed that workers must be heard before employers, ensuring a balanced dialogue. RTWCs also stressed the importance of power balance, although one said that it was sometimes hard to stay focused on the worker and achieve a power balance at meetings, continuing that:

I felt afterwards that she [the worker] became more and more silent and, yeah, no, we pushed too hard then. We had too much consensus over her head.

Thus, workers being the focus and being actively involved in collaboration was deemed to be fundamental in order to reach a genuine consensus.

The most prominent competing goal regarding a supportive environment was seen in several stakeholders stating that GPs obstruct RTW collaboration by protecting workers from SSIA investigations, and describing the RTW process as being too rapid. This became a dilemma for employers, who have a legal responsibility to speed up the RTW process [35]. One OHP said GPs’ tendency to overstate the barriers and only take the worker’s side created a bad environment at collaborative meetings. RTWCs, on the other hand, described hesitance among managers against work adjustments as related to managers’ goal to secure an adequate work environment for the worker’s colleagues, who otherwise might risk being overloaded and at risk of future sick leave as well.

#### Roles and responsibilities

Many stressed the shared role of working together and sharing the responsibility for a sustainable RTW process. When talking about stakeholders’ roles in achieving a supportive environment, RTWCs were mentioned as having a counselling and therapeutic role for workers. The workers described RTWCs as supporting them to be less anxious and offering *“a hand to hold on the way back to work”*, and as someone who advocated for them. Feeling trust in involved professionals, especially the RTWCs, was stressed as being crucial. RTWCs said they strived to gain trust from workers and that workers felt that encounters with the RTWCs were a secure spot. They also described their role as supporting, guiding, coaching and preparing workers before collaborative meetings.

Several stakeholders considered RTWCs to be central in creating a supportive environment, as they do not appear as a threat to the worker. One SSIA officer said this made RTWCs capable of pushing the RTW process forwards:

Often, I believe they maybe even run the case much better than us, because they get more in-depth knowledge about the patient and may not feel threatening, like an authority does. So, sometimes when you talk to the coordinator, you hear they have the same ideas […]. They’re two or three steps ahead of us.

RTWCs described their role as to defuse loaded situations that hinder a supportive environment and to promote power balance and a neutral collaborative arena where the worker’s perspective is in focus. Other stakeholders also stressed the importance of staying neutral, as expressed by OHPs and one HR specialist, since they should support both employers and workers. One OHP argued, however, that they work on behalf of the employer while the primary healthcare centre works on behalf of the worker.

Managers were sometimes described as not wanting or being able to take responsibility for providing necessary support. RTWCs promoted a supportive environment for managers, facilitating their decision-making and helping them write good rehabilitation plans. GPs also discussed that they could strengthen managers’ sense of security about how to act when fulfilling their role in explaining the medical status.

RTWCs had an important role in easing GPs’ workloads in collaboration and regarding rehabilitation issues. The GPs talked about the inherent role conflict between having a therapeutic alliance with their patient and being the medical expert, making decisions on whether or not to issue sick leave certification. The GPs discussed workers and employers wanting them to issue sick leave certificates, despite there being no medical reason for this. When RTWCs were the main contact at primary healthcare centres, this helped the GPs to distance themselves from such pressures. At the same time, the GPs discussed their responsibility to support the patient, even when they decided not to issue a certificate. It is clear that when the patient’s goal was to be granted further sickness certification rather than returning to work, the GP’s role became difficult. In other words, competing goals may lead to conflicting roles.

## Discussion

This study explored how the stakeholders involved describe the goals of the collaboration in the RTW process and how they experience their and other stakeholders’ roles and responsibilities in relation to the goals. The analysis identified shared and competing goals and different views on stakeholders’ roles and responsibilities that related to three important themes: creating an informative environment, striving for consensus in an environment of negotiations and creating a supportive environment. These three themes of environments were found to be central to collaboration in the RTW process for people with common mental disorders.

The main shared goals of RTW collaboration were improved communication, finding consensus about various issues in collaboration, working together on forward-looking goals, solving barriers and creating a supportive environment, especially for workers. The competing goals mostly concerned stakeholders’ different priorities, e.g., when to collaborate, on what and with whom, constituting an environment of negotiations. Similarly to previous research [8–10], competing goals and priorities were identified as hindering a successful collaboration. For example, if workers had other prioritised goals than returning to work, stakeholders renegotiated their role in collaboration, with some even stating that they should not be involved at all. Shared and clear goals were crucial for collaboration to be perceived as effective (cf. Young et al. [3]). It seems to be important that shared goals are not only discussed at collaborative meetings, but also articulated and written during the meeting, as there has found that one stakeholder otherwise writes goals after meetings, without having confirmed that this was the shared belief [39]. An important overarching goal with RTW collaboration is therefore to jointly formulate the specific goals in a worker’s RTW process, the RTW plan and the measures taken. This overarching goal might have the potential to make communication more concrete and effective, as it can avoid misunderstandings and further delays.

The goals in the sub-theme of when to collaborate incorporated different views of when it was deemed effective or efficient to participate in collaboration. SSIA officers hesitated to participate in early collaboration meetings, as others wanted them to, which can be understood as they were compliant with the SSIA’s organisational goal of using officers’ time effectively. Not prioritising participation in meetings can be understood as what Young et al. [3] refer to as a better ‘net benefit’ for the SSIA. However, the lack of flexibility to attend meetings made it harder for stakeholders to have concordant information, negotiate and reach a consensus. The fact that SSIA representatives showed a well-prepared united face, as described by Ahrne [27], involved an organisational strength and power position that other stakeholders did not possess in this negotiative environment. Fully aware that they were deemed as inflexible and having a decisive power, SSIA officers strived to show a softer side when they attended meetings (cf. Ahrne [27]) by communicating their goodwill, which previous research has outlined as important for a successful RTW process [5, 7]. The power imbalance, however, involved uncertainty for other stakeholders and difficulties in forming trustful relationships. Creating a supportive environment was closely associated with neutralising power imbalances, and our findings support those of Strömbäck et al. [23], showing that the RTWC have an important role in providing the support needed to balance unequal power relations in collaboration. Our study adds that the RTWC was considered to have a central function for workers with common mental disorders to feel trust in the involved stakeholders. Trust between the stakeholders involved in RTW collaboration is essential for clear and supportive communication [12, 13].

Creating an informative environment was an important shared goal, including information sharing, improved communication, clarifications and open and honest conversation. These aspects might be of particular importance when collaborating around workers with common mental disorders, as it can be emotionally difficult for a worker to talk about mental ill health, because this may include feelings of self-blame, stigmatisation and vulnerability [23, 40, 41]. Common mental disorders involve invisible symptoms and barriers which the worker needs to share with the other stakeholders so they can understand the workers’ situation, the stressors in the workplace environment and how to address them [41]. The invisibility and the sensitive character of mental health symptoms also put demands on the stakeholder’s information sharing, where each stakeholder has different insights that must be shared to achieve holistic considerations [40].

Consistent with findings from previous studies [14], the importance of clear roles and expectations in RTW collaboration was highlighted. However, our analysis showed a lack of consensus on how stakeholders perceived their roles and responsibilities. The RTWCs were deemed to have an important role in coordinating and enhancing trustful relationships between the involved stakeholders, and facilitating a supportive environment and power balance in collaboration (cf. Ahrne [27]). In line with other studies about RTWC in Swedish primary healthcare [11, 24–26, 32, 42], we found that stakeholders perceive their contribution to RTW collaboration to be valuable, and they expressed high expectations of the RTWC role. However, RTWCs themselves described their role and responsibilities in a way that differs from other stakeholders. Furthermore. it has been shown in other studies as well that they perceive their role differently [21] and use different work models with regard to whether they select patients and to what extent they choose to collaborate with stakeholders [22].

Similarly to the scoping review of Corbière et al. [2], the RTWCs were ascribed an informative role, being forward-looking, making contact, proposing and preparing for meetings, monitoring and adjusting RTW plans, carrying out follow-ups, supporting workers and promoting an open, honest and safe environment at meetings. Further, the RTWCs were often deemed flexible, reflecting the fact that their assignment, role and responsibilities were not clearly regulated. Flexibility is suggested to facilitate negotiations and power balance in collaboration [27], but also meant that some RTWCs negotiated their boundaries regarding whom they should collaborate around [28], e.g., by refusing cases that did not correspond with their own goals of early collaboration in sick leave cases. Previous studies have raised the question of how RTWCs exclude workers as imposing a risk of unequal access, and is a potential ethical issue in RTW coordination [21, 32]. The overall goal of early collaboration is found to be important for the RTW process. It is also important, however, that workers are not denied support and collaboration when needed, if the goal of early collaboration has failed.

**Fig. 1:**
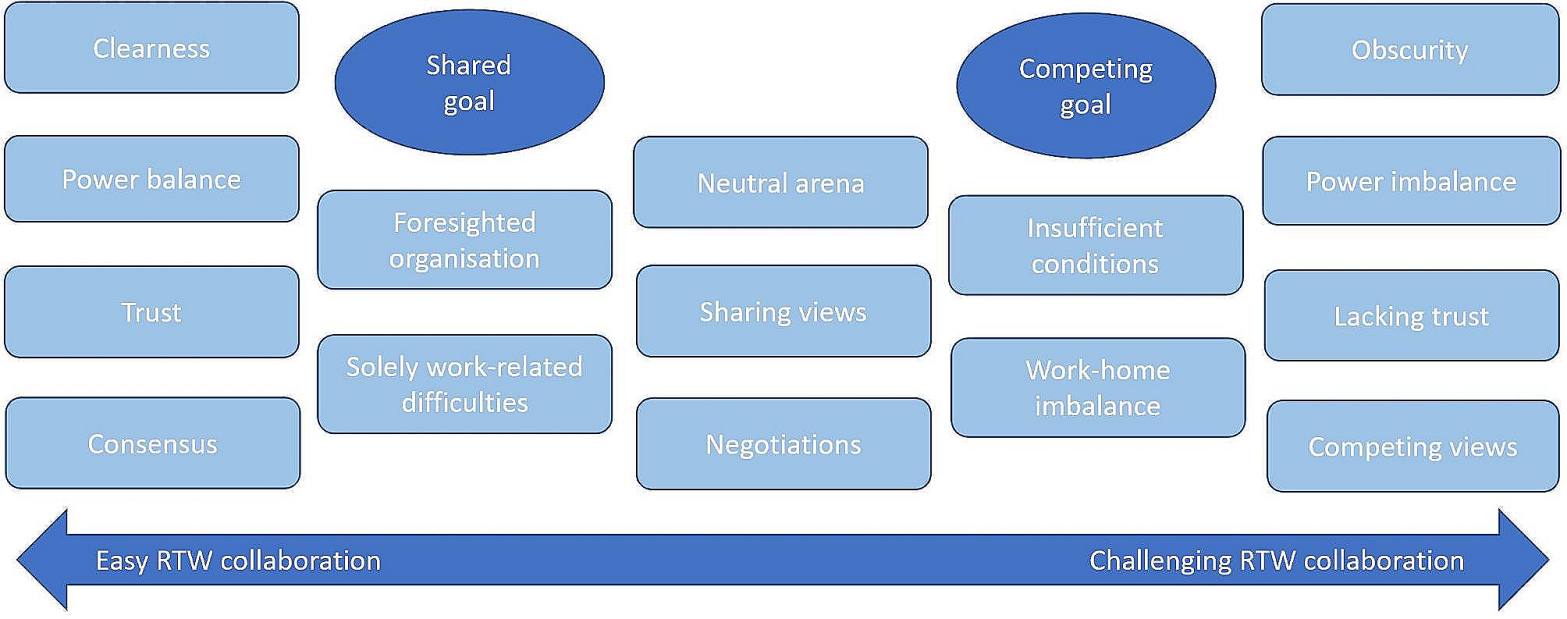
Conditions for easy versus challenging return-to-work (RTW) collaboration RTW = return-to-work

This study outlines how collaboration involves tensions that should be considered. There were overall patterns in the results, as presented in Fig. 1, which shows a spectrum between easy versus challenging RTW collaboration. The easy collaboration involved shared goals that made all stakeholders strive in the same direction and guided action in the RTW process. This was related to clear communication, goal setting, and a collaborative space characterised by mutual trust and a power balance where the worker could be honest without fearing consequences. At the challenging end of the spectrum, competing goals and priorities led stakeholders in different directions, which obstructed consensus in RTW collaboration. Competing goals were related to obscurity in communication, competing views, lack of stakeholder trust, and power imbalances. Other conditions affecting collaboration concerned whether there were organisational policies or routines for when and how to collaborate, which either created insufficient conditions for collaboration, or contributed to the foresight of organisations to create effective and efficient collaboration. The findings suggest some basic conditions for achieving shared goals and consensus with clear communication and roles; a neutral collaborative arena for sharing views and where negotiations can occur.

Figure 1 should not be interpreted as suggesting that easy collaboration is always ideal for all situations – societies are full of stakeholders with different roles, focusing on their specific assignments and interests. Gray [43] describes collaboration not as downplaying self-interest or as the end of the conflict, but rather as constructive management of differences. Understood this way, consensus may not be the most important goal of collaboration; it can rather involve creating a collaborative arena that is neutral in terms of power balance, where all stakeholders can share their different views and negotiate on the problem description, problem-solving and resource allocation.

In post-pandemic times, it is interesting to consider the forms of collaboration, onsite or remotely. The interviews were made before the COVID-19 pandemic outbreak, and the question of remote collaboration was not brought up. We do know, however, that it was common for healthcare personnel in a meeting with workers on sick leave to have telephone conferences with for example SSIA officers, so remote collaboration techniques were not new. It is likely, however, that remote forms of collaboration have increased, due to more people being used to video meetings today, and that online meetings offer flexibility. To the best of our knowledge, there are no studies about what impact remote meetings may have on RTW collaboration, but one integrative review of the consequences of visiting restrictions during the COVID-19 pandemic showed that remote meetings, compared to meetings at hospital wards, can reduce the possibility of providing consensus-based care [44]. Studies on the positive and negative aspects of remote collaboration techniques are warranted.

## Methodological considerations

Our qualitative approach, with interview data from a range of stakeholders, helps us to understand factors that link and differentiate stakeholders in RTW collaboration. This adds to a research field where most studies include only a few of the involved stakeholders, and gives holistic and nuanced knowledge.

We conducted a secondary analysis of an emerging issue that the participants addressed during the semi-structured interviews [30, 35]. In any secondary analysis, the study aim must be relevant and meaningful to address the data used, if the data was not collected with the specific study aim in mind [35]. In this study, some interview questions specifically addressed roles in coordination and rich data about roles and goals of collaboration were also identified in response to other questions in the semi-structured interview. This way, the interviews generated rich data for secondary analyses on questions that were either directly, or indirectly addressed in the interview guides.

Although this large dataset consists of 41 individual interviews and one focus group interview with five individuals, there is some imbalance in the representation of stakeholders, with few [4–6] individuals representing some of the stakeholder groups, and a large group of RTWCs [18]. This means that the data consisted of thick descriptions from the RTWCs, and less thick descriptions from other stakeholder groups. A strategy to handle this imbalance was to analyse and present each stakeholder group separately, so that the views of the smaller groups were acknowledged. It is possible that a more balanced representation of stakeholders would have generated a different result.

The fact that the GPs were interviewed as a group and thus had a joint intra-professional discussion may have influenced the results. For example, they focused more on their own work and roles in their group discussion, compared to other stakeholders in the individual interviews. Of those who participated in individual interviews, 18 chose to be interviewed by telephone, and notably, seven out of nine workers. Worries have been raised that telephone interviews involve a negative impact on the richness and quality of collected data, and the possibilities to respond to visual and emotional cues [45] – but a growing body of studies does not confirm such worries [34, 46–48]. In a recent study where researchers asked 32 people with common mental disorders about being interviewed by mobile phone, participants experienced less demands in terms of emotion work and social responsibility in the interview situation [31]. For some, the flexibility provided by the telephone option was the stated reason for deciding to participate in the study, indicating that this data collection method results in more people being allowed to share their experiences with sensitive issues [34, 49]. However, the loss of visual communication requires more active listening and use of vocalisations and clarifications [49], as well as being prepared for difficulties with technology and disturbing noise from the surroundings [31]. There were no technological or noise disturbances affecting the data collected through telephone, and consistent with previous research [50] we did not identify differences in the richness and depth of participants’ stories between telephone and face-to-face interviews. It is argued that telephone interviews can be regarded as a valuable first option if the purpose of the study is to listen to people’s experiences, and not to observe visual cues [34].

The validity and trustworthiness of the results were strengthened by the fact that six authors who were familiar with the data were involved in different steps of the research process, and discussed the analysis and interpretation of the results. As with most qualitative studies, one should be cautious about generalising based on the results. The outlined patterns in Fig. 1 should be understood as conceptual or theoretical suggestions that contribute to a degree of generalisability [36], but that need to be studied in more depth in the future. However, as some findings align with previous studies, the results can be regarded as bricks added to the cumulative knowledge of various stakeholders’ views on collaboration goals in the RTW process.

The transferability of research within the field of vocational rehabilitation is often limited by the variation in culture, welfare systems and policies between countries. The description of the Swedish sickness insurance model in this study can guide the assessment of the transferability to other settings and populations of experiences regarding RTW collaboration on people on sick leave due to common mental disorders.

## Conclusions

RTW collaboration is generally supposed to clarify responsibilities, enhance communication, and make communication more efficient. The findings of this study raise the question of whether the collaboration itself – and consequently the communication – could be more efficient if the goals of the collaboration are clarified. This study contributes new knowledge about the shared and competing goals of the collaboration among six various stakeholders involved in the RTW process for workers with common mental disorders, and how such goals relate to expected roles and responsibilities among them. In line with previous research, competing goals and priorities of different stakeholders were identified as a barrier to successful collaboration – an aspect that should be considered when stakeholders set their own goals and priorities. The study presents a figure that outlines certain aspects of the collaborative environment that lead to easy versus challenging RTW collaboration which could guide the practice in the consideration of the goals of the collaboration. RTWCs are found to have an important role in facilitating an informative and supportive collaborative environment, and in neutralising power imbalances between workers and their employers and SSIA officers.

## Data Availability

The data is available upon reasonable request and after ethical approval from the Swedish Ethical Review Authority.

## Abbreviations

ER: Employer representative
GP: General practitioner
OHP: Occupational health professional
RTW: Return-to-work
RTWC: Return-to-work coordinator
SSIA: the Swedish social insurance agency

## References

[1] Lindqvist R (2003). Vocational rehabilitation between work and welfare-the Swedish experience. Scandinavian J Disabil Res.

[2] Corbière M, Mazaniello-Chézol M, Bastien M-F, Wathieu E, Bouchard R, Panaccio A (2020). Stakeholders’ role and actions in the return-to-work process of workers on sick-leave due to common mental disorders: a scoping review. J Occup Rehabil.

[3] Young AE, Wasiak R, Roessler RT, McPherson KM, Anema J, Van Poppel MN (2005). Return-to-work outcomes following work disability: stakeholder motivations, interests and concerns. J Occup Rehabil.

[4] Higgins A, O’Halloran P, Porter S (2012). Management of long term sickness absence: a systematic realist review. J Occup Rehabil.

[5] Pransky G, Katz JN, Benjamin K, Himmelstein J (2002). Improving the physician role in evaluating work ability and managing disability: a survey of primary care practitioners. Disabil Rehabil.

[6] Andersen MF, Nielsen KM, Brinkmann S (2012). Meta-synthesis of qualitative research on return to work among employees with common mental disorders. Scand J Work Environ Health.

[7] MacEachen E, Clarke J, Franche RL, Irvin E (2006). Systematic review of the qualitative literature on return to work after injury. Scand J Work Environ Health.

[8] Eriksson UB, Engstrom LG, Starrin B, Janson S (2008). Falling between two stools; how a weak co-operation between the social security and the unemployment agencies obstructs rehabilitation of unemployed sick-listed persons. Disabil Rehabil.

[9] Skivington K, Lifshen M, Mustard C (2016). Implementing a collaborative return-to-work program: lessons from a qualitative study in a large Canadian healthcare organization. Work.

[10] Ståhl C, Svensson T, Petersson G, Ekberg K (2010). A matter of trust? A study of coordination of Swedish stakeholders in return-to-work. J Occup Rehabil.

[11] Holmlund L, Hellman T, Engblom M, Kwak L, Sandman L, Törnkvist L (2022). Coordination of return-to-work for employees on sick leave due to common mental disorders: facilitators and barriers. Disabil Rehabil.

[12] Loisel P, Durand M-J, Baril R, Gervais J, Falardeau M (2005). Interorganizational collaboration in occupational rehabilitation: perceptions of an interdisciplinary rehabilitation team. J Occup Rehabil.

[13] Corbière M, Mazaniello-Chézol M, Lecomte T, Guay S, Panaccio A (2022). Developing a collaborative and sustainable return to work program for employees with common mental disorders: a participatory research with public and private organizations. Disabil Rehabil.

[14] Russell E, Kosny A (2019). Communication and collaboration among return-to-work stakeholders. Disabil Rehabil.

[15] MacEachen E, McDonald E, Neiterman E, McKnight E, Malachowski C, Crouch M et al. Return to work for mental ill-health: a scoping review exploring the impact and role of return-to-work coordinators. J Occup Rehabil. 2020:1–11. 10.1007/s10926-020-09873-3 PMC7406484 32002709

[16] Vogel N, Schandelmaier S, Zumbrunn T, Ebrahim S, de Boer W, Busse J (2017). Return-to‐work coordination programmes for improving return to work in workers on sick leave. Cochrane Libr.

[17] Franche R-L, Cullen K, Clarke J, Irvin E, Sinclair S, Frank J (2005). Workplace-based return-to-work interventions: a systematic review of the quantitative literature. J Occup Rehabil.

[18] Schandelmaier S, Ebrahim S, Burkhardt SC, de Boer WE, Zumbrunn T, Guyatt GH (2012). Return to work coordination programmes for work disability: a meta-analysis of randomised controlled trials. PLoS ONE.

[19] Corbière M, Mazaniello-Chézol M, Bastien MF, Wathieu E, Bouchard R, Panaccio A, Guay S, Lecomte T (2020). Stakeholders’ role and actions in the return-to-work process of workers on sick-leave due to common mental disorders: a scoping review. J Occup Rehabil.

[20] Dol M, Varatharajan S, Neiterman E, McKnight E, Crouch M, McDonald E et al. Systematic review of the impact on return to work of return-to-work coordinators. J Occup Rehabil. 2021:1–24. 10.1007/s10926-021-09975-6 33881671

[21] Svärd V, Jannas S (2023). Organisational prerequisites for coordinating the return-to-work process for people with multimorbidity and psychosocial difficulties. Disabil Rehabil.

[22] Svärd V, Berglund E, Björk Brämberg E, Gustafsson N, Engblom M, Friberg E (2023). Coordinators in the return-to-work process: mapping their work models. PLoS ONE.

[23] Strömbäck M, Fjellman-Wiklund A, Keisu S, Sturesson M, Eskilsson T (2020). Restoring confidence in return to work: a qualitative study of the experiences of persons with exhaustion disorder after a dialogue-based workplace intervention. PLoS ONE.

[24] Svärd V, Friberg E, Azad A (2021). How people with multimorbidity and psychosocial difficulties experience support by rehabilitation coordinators during sickness absence. J Multidiscipliniary Healthc.

[25] Azad A, Svärd V (2021). Patients’ with Multimorbidity and Psychosocial difficulties and their views on important Professional competence for Rehabilitation coordinators in the return-to-work process. Int J Environ Res Public Health.

[26] Berglund E, Friberg E, Engblom M, Andersén Å, Svärd V (2022). Coordination and perceived support for return to work: a cross-sectional study among patients in Swedish Healthcare. Int J Environ Res Public Health.

[27] Ahrne G (1994). Social organizations. Interactions inside, outside and between organizations.

[28] Langley A, Lindberg K, Mørk BE, Nicolini D, Raviola E, Walter L (2019). Boundary work among groups, occupations, and organizations: from cartography to process. Acad Manag Ann.

[29] Braun V, Clarke V (2006). Using thematic analysis in psychology. Qualitative Res Psychol.

[30] Braun V, Clarke V (2022). Conceptual and design thinking for thematic analysis. Qualitative Psychol.

[31] Björk Brämberg E, Sandman L, Hellman T, Kwak L (2019). Facilitators, barriers and ethical values related to the coordination of return-to-work among employees on sick leave due to common mental disorders: a protocol for a qualitative study (the CORE-project). BMJ open.

[32] Holmlund L, Sandman L, Hellman T, Kwak L, Björk Brämberg E. Ethical aspects of the coordination of return-to-work among employees on sick leave due to common mental disorders: a qualitative study. Disabil Rehabil. 2022:1–10. 10.1080/09638288.2022.2084779 35676198

[33] Tong A, Sainsbury P, Craig J (2007). Consolidated criteria for reporting qualitative research (COREQ): a 32-item checklist for interviews and focus groups. Int J Qual Health Care.

[34] Azad A, Sernbo E, Svärd V, Holmlund L, Björk Brämberg E (2021). Conducting In-Depth interviews via Mobile Phone with persons with Common Mental disorders and Multimorbidity: the challenges and advantages as experienced by participants and researchers. Int J Environ Res Public Health.

[35] Chatfield SL (2020). Recommendations for secondary analysis of qualitative data. Qualitative Rep.

[36] Thompson J (2022). A guide to abductive thematic analysis. Qualitative Rep.

[37] The Swedish Work Environment Authority (2020). Job modification: the Swedish Work Environment Authority’s provisions and general recommendations concerning job modification.

[38] The Swedish Association of Local Authorities and Regions (SALAR). Metodbok för koordinering. [Handbook for RTW coordination]. 2020:1–180.

[39] Poulsen RM, Hoffmann Pii K, Bültmann U, Meijer M, Falgaard Eplov L, Albertsen K, Christensen U (2019). Developing normative integration among professionals in an intersectoral collaboration: a Multi-method Investigation of an Integrated intervention for people on sick leave due to Common Mental disorders. Int J Integr Care.

[40] Meling HM, Anderssen N, Ruths S, Hjörleifsson S, Haukenes I (2023). Stakeholder views on work participation for workers with depression and intersectoral collaboration in depression care: a focus group study with a salutogenic perspective. Scand J Prim Health Care.

[41] Scharf J, Angerer P, Müting G, Loerbroks A (2020). Return to work after common mental disorders: a qualitative study exploring the expectations of the involved stakeholders. Int J Environ Res Public Health.

[42] Berglund E, Friberg E, Engblom M, Svärd V. Physicians’ experience of and collaboration with return-to-work coordinators in healthcare: a cross-sectional study in Sweden. Disabil Rehabil. 2023:1–9. 10.1080/09638288.2023.2261851 37772755

[43] Gray B, Collaborating (1989). Finding Common Ground for Multiparty problems.

[44] Hugelius K, Harada N, Marutani M (2021). Consequences of visiting restrictions during the COVID-19 pandemic: an integrative review. Int J Nurs Stud.

[45] Shuy RW, Gubrium JF, Holstein JA (2002). In-person versus telephone interviewing. Handbook of interview research: Context and Method.

[46] Ward K, Gott M, Hoare K (2015). Participants’ views of telephone interviews within a grounded theory study. J Adv Nurs.

[47] Cachia M, Millward LJ (2011). The telephone medium and semi-structured interviews: a complementary fit. Qualitative Res Organ Manage.

[48] Holt A (2010). Using the telephone for narrative interviewing: a research note. Qualitative Res.

[49] Drabble L, Trocki KF, Salcedo B, Walker PC, Korcha RA (2016). Conducting qualitative interviews by telephone: lessons learned from a study of alcohol use among sexual minority and heterosexual women. Qualitative Social Work.

[50] Vogl S (2013). Telephone versus face-to-face interviews: Mode effect on semistructured interviews with children. Sociol Methodol.

